# First Molecular Detection of *Babesia gibsoni* in Dogs from Wuhan, China

**DOI:** 10.3389/fmicb.2017.01577

**Published:** 2017-08-21

**Authors:** Lan He, Xiaoyan Miao, Jinfang Hu, Yuan Huang, Pei He, Junwei He, Long Yu, Ngabu Malobi, Ligang Shi, Junlong Zhao

**Affiliations:** ^1^State Key Laboratory of Agricultural Microbiology, College of Veterinary Medicine, Huazhong Agricultural University Wuhan, China; ^2^Key Laboratory for Development of Veterinary Diagnostic Products, Ministry of Agriculture, Huazhong Agricultural University Wuhan, China; ^3^Luoyang Center for Animal Disease Control and Prevention Luoyang, China

**Keywords:** *Babesia gibsoni*, babesiosis, reverse line blot, 18S rRNA, pet dog, companion animal

## Abstract

Canine piroplasmosis is a significant disease in dogs caused by *Babesia* and *Theileria* parasites. The clinical manifestations range from mild illness to serious disease depending on the parasite species and the physical condition of the infected dog. Canine piroplasmosis has been reported to be prevalent in China. However, no molecular evidence of the disease has been reported in pet dogs from Wuhan. In this study, 118 blood samples were randomly collected from pet dogs in veterinary clinics. The blood samples were subjected to both microscopic examination and reverse line blot (RLB) hybridization assays to detect piroplasm infection. Parasites were observed in 10 blood samples via microscopic examination, whereas there were 14 *Babesia gibsoni*-positive RLB tests. Phylogenetic analysis was performed after the 18S rRNA and ITS gene sequences from the 14 positive samples were cloned and sequenced. The results confirmed the existence of *B. gibsoni* in this area. This is the first molecular report of canine babesiosis in pet dogs from Wuhan, China. Pet dogs are companion animals, and the prevalence of babesiosis will be of concern in daily life. This study will help veterinarians better understand the prevalence of canine babesiosis and provide a guide for disease control in pet dogs.

## Introduction

Piroplasmosis is a serious disease caused by an intracellular hemoprotozoan with a worldwide distribution. It can infect animals as well as humans (Service, 2001; Solano-Gallego and Baneth, 2011; Schnittger et al., 2012). Canine piroplasmosis is one of the most important tick-borne infectious diseases. It is now regarded as a common and significant disease of dogs because several different species have been identified (Yisaschar-Mekuzas et al., 2013). The clinical manifestations range from mild illness to serious disease, depending on the infecting parasite species and the nutritional status, age, and immune condition of the dog (Muhlnickel et al., 2002; Schoeman, 2009; Schnittger et al., 2012). Typical symptoms include fever, anemia, pallor, jaundice, hemoglobinuria, splenomegaly, and weakness (Beck et al., 2009; Bajer et al., 2014).

There are eight *Babesia* and *Theileria* species that infect canines, which are classified as four large and four small species (Kjemtrup et al., 2006; Schoeman, 2009; Yisaschar-Mekuzas et al., 2013). The large species are *Babesia canis vogeli, Babesia canis canis, Babesia canis rossi*, and *Babesia* sp. (unnamed) which was identified in dogs in North Carolina (Zahler et al., 1998; Carret et al., 1999; Irwin, 2009). *B. gibsoni* is a small piroplasm that is distributed worldwide. Three other small piroplasms are *Babesia conradae, Babesia vulpes*, and *Theileria* sp. (unnamed; Kjemtrup et al., 2006; Matjila et al., 2008; Baneth et al., 2015). As tick-transmitted parasites, the prevalence of piroplasmosis depends on the distribution of the transmitted tick vectors. However, dog bites, blood transfusions, and transplacental transmission may represent alternative routes of transmission (Fukumoto et al., 2005; Vichova et al., 2014).

According to the records of the Chinese Center for Disease Control and Prevention, there were 130 million dogs in China in 2012. In Wuhan city, the human population is more than 12,000,000, and there are ~1,000,000 dogs. The south and east regions of China are the most endemic regions for these parasites (Wei et al., 2012; Chen et al., 2014). In Shanghai, the seroprevalence of *B. gibsoni* was determined to be 9.23% via indirect ELISA (Cao et al., 2015). Yao et al. reported that *B. gibsoni* is the main species responsible for canine babesiosis in Nanjing (Yao et al., 2014). In Jiangxi, the rates of positivity for *B. canis vogeli* and *B. gibsoni* are 4.94 and 2.47%, respectively, as tested by species-specific PCR (Zheng et al., 2017). In 2017, Niu et al. first reported the identification of *Theileria sinensis* in pet dogs from Gansu province of China, providing the first report of *T. sinensis* in dogs worldwide (Niu et al., 2017).

However, there have been no reports describing canine piroplasmosis in Wuhan, China. Therefore, the aim of this study was to investigate the occurrence of piroplasma infection in pet dogs in Wuhan.

## Materials and methods

### Sample collection

According to official information from the Wuhan Animal Health Inspection Institute, China, there are 89 veterinary clinics and hospitals in Wuhan, China. In the present study, a total of 118 blood samples from pet dogs were randomly collected from five clinics with the permission of the dogs' owners. All samples were screened via both microscopic examination and reverse line blot (RLB) hybridization assays at the College of Veterinary Medicine of Huazhong Agricultural University.

### DNA extraction

Genomic DNA was extracted from 200 μl of EDTA anti-coagulated blood using the TIANamp Genomic DNA Kit (TransGen Biotech, Beijing, China) according to the manufacturer's instructions. The concentrations of the extracted DNA were measured with a NanoDrop 2000 (Thermo Scientific, USA). The isolated DNA samples were used immediately or stored at −20°C.

### Reverse line blot hybridization assay

A pair of primers, RLB-F2 (5′-GAC ACA GGG AGG TAG TGA CAA G-3′) and RLB-R2 (5′-biotin-CTA AGA ATT TCA CCT CTG ACA GT-3′; Nijhof et al., 2003, 2005), was used to amplify the V4 variable region of the 18S rRNA gene of both *Babesia* and *Theileria*. Touchdown PCR was performed in a total volume of 25 μl, containing 2.5 μl of 10 × PCR buffer, 2 μl of 2.5 mM dNTP Mixture, 0.1 μM each primer, 0.3 μl of 5 U/μl r*Taq* polymerase (Takara Biotechnology, China), 2.5 μl of extracted genomic DNA, and double distilled water. As positive and negative controls, we used genomic DNA from *B. orientalis* that was stored in our laboratory and RNase-free water, respectively.

Oligonucleotide probes (Table 1) containing an N-terminal N-(trifluoracetamidohexyl-cyanoethyl, *N,N-*diisopropyl phosphoramidite [TFA])-C6 amino linker were synthesized by Augct (Beijing, China). Six known canine piroplasms (*B. canis, B. vogeli, B. rossi, B. gibsoni, B. conradae*, and *B. vulpes*) and related *Babesia* and *Theileria* species were targeted by these probes. An RLB hybridization assay was then conducted as previously described (Gubbels et al., 1999). Briefly, a Biodyne C membrane was activated at room temperature using 16% (wt/wv) 1-ethyl-3-(3-dimethyl-amino-propyl) carbodiimide (EDAC) (Sigma, USA) for 10 min, after which the oligonucleotide probes were covalently linked to the membrane at optimal concentrations (Table 1) in 0.5 M NaHCO_3_ for 1 min in a miniblotter. The membrane was subsequently inactivated by 100 mM NaOH for 8 min after washing in 2 × SSPE/0.1% SDS at 60°C for 5 min and then either directly used or stored at 4°C in 20 mM EDTA, pH 8.0. For the assays, 10 μl of PCR product was added to 140 μl of 2 × SSPE/0.1% SDS after denaturing at 100°C for 10 min, followed by immediate cooling on ice. The denatured PCR products were then added to the miniblotter, which was a pre-prepared Biodyne C membrane, and hybridized at 42°C for 60 min. The membrane was subsequently washed twice in preheated 2 × SSPE/0.5% SDS at 50°C for 10 min, incubated for 30 min at 42°C in 2 × SSPE/0.5% SDS with 2.5 μl of streptavidin-POD conjugate (Roche Diagnostic, Germany), washed twice in preheated 2 × SSPE/0.5% SDS at 42°C for 10 min, and finally washed twice in 2 × SSPE for 5 min at room temperature. Hybridization detection was performed using chemiluminescence.

**Table 1:** Oligonucleotide RLB probes used in the study and their references.

**Probe**	**Sequence (5′-3′)**	**Concentration (pmol)**	**References**
*Babesia canis vogeli*	AGC GTG TTC GAG TTT GCC	200	Matjila et al., 2008
*Babesia gibsoni*	CAT CCC TCT GGT TAA TTT G	200	Matjila et al., 2007
*Babesia canis canis*	TGC GTT GAC CGT TTG AC	200	Matjila et al., 2008
*Babesia canis rossi*	CGG TTT GTT GCC TTT GTG	100	Matjila et al., 2008
*Babesia conradae*	CGT TCC CTT CGG GGC	200	Yisaschar-Mekuzas et al., 2013
*Babesia orientalis*	CCT CTT TTG GCC GTC TCA CT	400	He et al., 2012
*Babesia occultans*	CCT CTT TTG GCC CAT CTC GTC	400	He et al., 2012
*Babesia bigemina*	CGT TTT TTC CCT TTT GTT GG	100	Gubbels et al., 1999
*Babesia ovis*	TGC GCG CGG CCT TTG CGT T	100	Schnittger et al., 2004
*Babesia bovis*	CAG GTT TCG CCT GTA TAA TTG AG	100	Gubbels et al., 1999
*Babesia* sp. *(xinjiang)*	GCG GGT TTC GTC TAC TTC GCT TTG T	400	He et al., 2012
*Babesia* sp. *(sable)*	GCG TTG ACT TTG TGT CTT TAGC	400	Oosthuizen et al., 2008
*Babesia microti*	GAC TTG GCA TCT TCT GGA	400	Nijhof et al., 2003
*Babesia crassa catch-all*	GTT GGC TTA TCT TTT TAC TTT	100	Schnittger et al., 2004
*Babesia genus-specific 1*	ATT AGA GTG TTT CAA GCA GAC	100	Bhoora et al., 2009
*Babesia genus-specific 2*	ACT AGA GTG TTT CAA ACA GGC	100	Bhoora et al., 2009
*Babesia/Theileria genus specific*	TAA TGG TTA ATA GGA RCR GTT G	100	Gubbels et al., 1999
*Theileria genus specific*	ATT AGA GTG CTC AAA GCA GGC	200	He et al., 2012
*Theileria lestoquardi*	CTT GTG TCC CTC CGG G	400	Schnittger et al., 2004
*Theileria taurotragi*	TCT TGG CAC GTG GCT TTT	400	Gubbels et al., 1999
*Babesia vulpes*	CTT ATC ATT AAT TTC GCT TCC GAA CG	400	Yisaschar-Mekuzas et al., 2013
*Theileria annulata*	CCT CTG GGG TCT GTG CA	400	Gubbels et al., 1999
*Theileria ovis*	TTG CTT TTG CTC CTT TAC GAG	400	Schnittger et al., 2004
*Theileria mutans*	CTT GCG TCT CCG AAT GTT	400	Gubbels et al., 1999
*Theileria orientalis 1*	GGC TTA TTT CGG ATG ATA CTT GT	400	He et al., 2012
*Theileria orientalis 2*	GGC TTA TTT CGG ATG ATA CTT GT	400	He et al., 2012
*Theileria* sp. *(buffalo)*	CAG ACG GAG TTT ACT TTG T	400	Oura et al., 2004
*Theileria buffeli*	GGC TTA TTT CGG WTT GAT TTT	400	Gubbels et al., 1999
*Theileria sinensis*	TCG CAT CTC TTG CTG AGT GC	400	He et al., 2012

### Cloning and sequencing of 18S rRNA genes and its regions

The partial 18S rRNA gene and the ITS region were amplified from 14 samples that tested positive via RLB using the primer pairs P1/P2 and ITSF/ITS2, respectively (Table 2). PCR amplification of the 18S rRNA gene and ITS sequences was performed in a total volume of 50 μl, with 10 μl of 5 × *TransStart FastPfu* Buffer, 5 μl of a 2.5 mM dNTP Mixture, 0.1 μM of each primer, 1 μl of *TransStart FastPfu* DNA Polymerase (Takara Biotechnology, Dalian, China), 2.5 μl of genomic DNA, and double distilled water. The conditions for PCR amplification of the 18S rRNA gene were as follows: an initial denaturation step at 95°C for 2 min; 35 cycles of denaturation for 20 s at 95°C, annealing for 20 s at 55°C, and extension for 45 s at 72°C; and a final extension step of 5 min at 72°C. The PCR amplification conditions for the ITS region were almost the same as for the 18S rRNA gene, except that the annealing temperature used was 54°C in this step. The PCR products were purified using the Easypure Quick Gel Extraction Kit (TransGen Biotech, Beijing, China). The purified amplicons were cloned into the pMD19-T vector (Takara Biotechnology, China), which was then transformed into *E. coli* JM109 cells (TaKaRa Biotechnology, China) according to the manufacturer's instructions. Three positive colonies of each sample were selected for sequencing (ABI PRISM 377 DNA sequencer).

**Table 2:** Primers used to amplify the 18S rRNA gene and ITS region.

**Primer**	**Sequence**	**Amplicon size (bp)**
P1	5′-AACCTGGTTGATCCTGCCAGTAGTCAT-3′	1,700
P2	5′-GAT CCT TCT GCA GGT TCA CCT AC-3′	
ITS F	5′-GAGAAGTCGTAACAAGGTTTCCG-3′	1,100
ITS 2	5′-ACAATTTGCGTTCAATCCCA-3′	

### Phylogenetic analysis

The obtained 18S rRNA and ITS sequences were subjected to BLAST analysis in the GenBank database. Multiple sequence alignment with related genes was conducted using MAFFT (version 7) (Katoh and Frith, 2012), and the alignment was edited with BioEdit (version 7.0.9). Phylogenetic trees based on the 18S rRNA and ITS nucleotide sequences were constructed using MEGA6 software (Tamura et al., 2013). All analyses were performed with 1,000 bootstrap replications.

### Ethics statement

This study was approved by the Scientific Ethic Committee of Huazhong Agricultural University (permit number HZAUDO-2014-006). All pet dogs were handled in accordance with the Animal Ethics Procedures and Guidelines of the People's Republic of China. All samples were collected under the permission of the pet dogs' owners.

## Results

### Microscopy and RLB results

**Figure 1:**
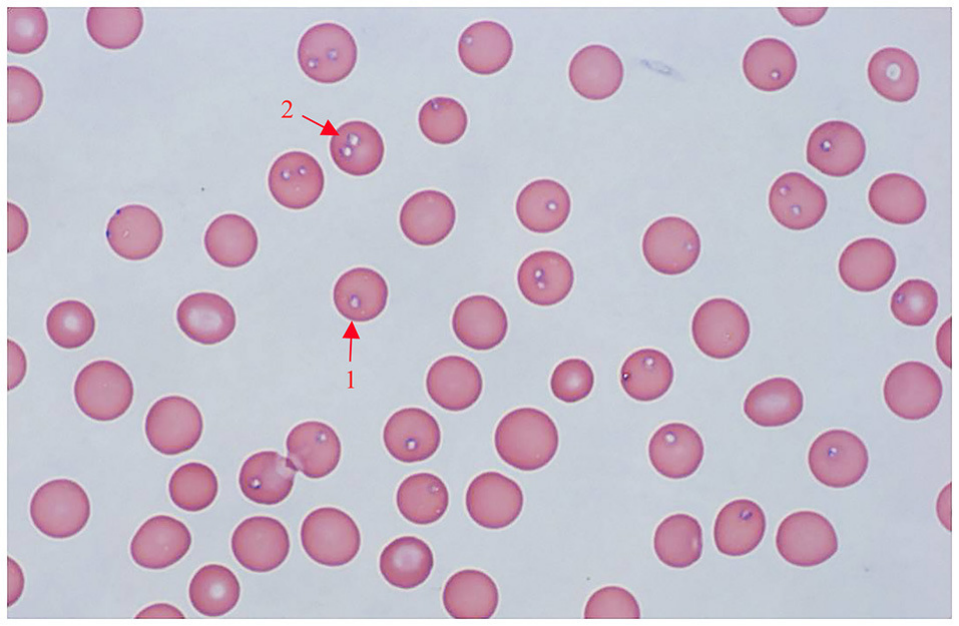
Giemsa-stained thin blood smear of *Babesia gibsoni* in dog erythrocytes. Final magnification is 1000X, oil. 1, Single pyriform; 2, double pyriform.

All samples (*n* = 118) were screened via both microscopy and RLB. *Babesia*-like parasites were observed in 10 samples through microscopic examination (Figure 1). The clinical records showed that those 10 dogs had fever, anemia, pallor, and even hemoglobinuria. Fourteen samples, including the 10 microscopy-positive samples, tested positive for *B. gibsoni* by RLB.

### 18S rRNA and its sequencing

The nucleotide sequences of the 18S rRNA and ITS genes obtained in this study were submitted to GenBank under accession numbers KP666155-KP666168 and KP666141-KP666154, respectively.

### Nucleotide sequence analysis

Blast analysis showed that the sequenced 18S rRNA genes shared a high identity of 99.2–99.9% with the 18S rRNA genes of *B. gibsoni* (DQ184507). The ITS sequences shared high identity with *B. gibsoni* (EU084673). Nucleotide sequence variations within the 18S rRNA and ITS gene sequences were observed. The identity of the obtained 18S rRNA sequences ranged from 99.4 to 100%, with a 0–10 bp difference. Additionally, the ITS genes obtained in this study showed 98.9–100% identity, with a 0–12 bp nucleotide difference.

### Phylogenetic analysis

**Figure 2:**
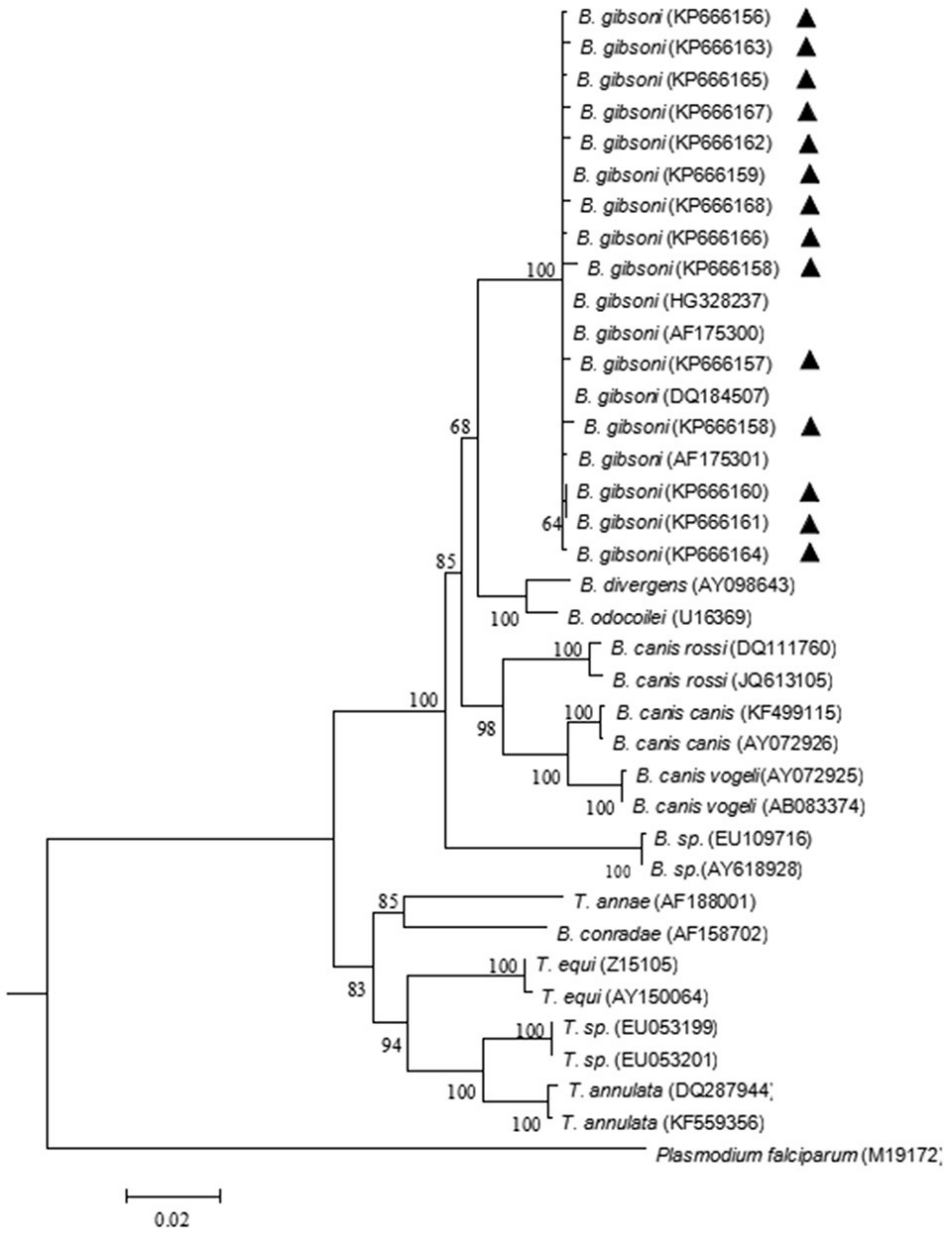
Neighbor-joining tree based on the full-length 18S rRNA gene sequences obtained in this study (▴) and the related sequences. The 18S rRNA of *Plasmodium falciparum* was employed as an outgroup. GenBank accession numbers are indicated in parentheses. A bootstrap test of 1,000 replicates was used, and values are given at the nodes.

**Figure 3:**
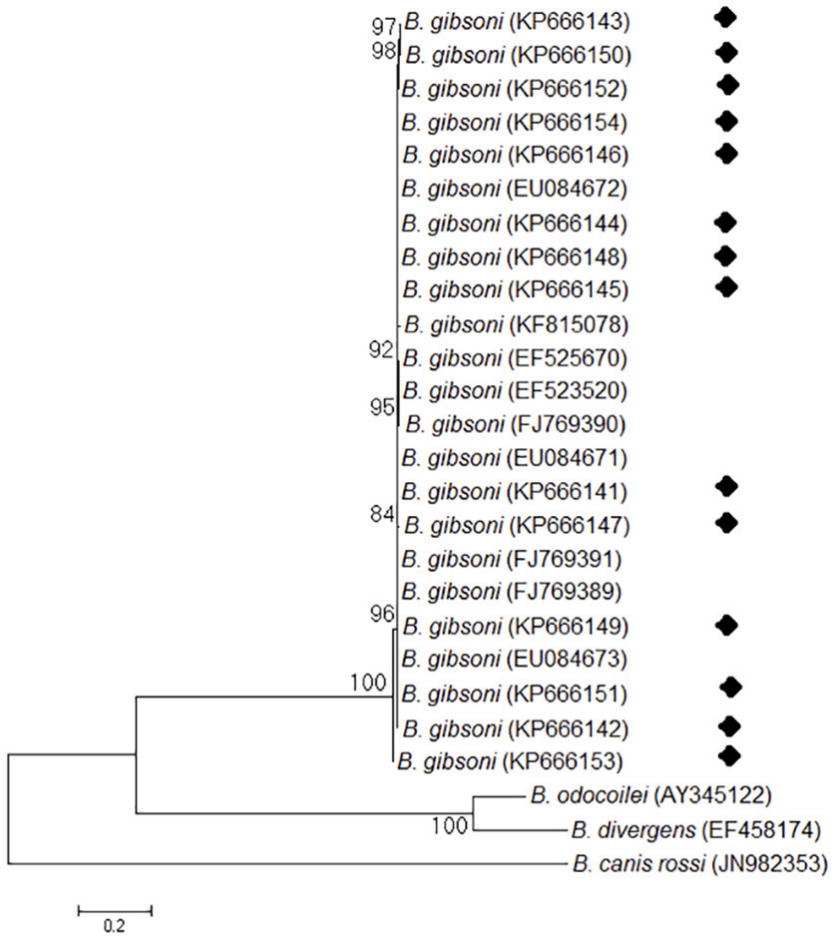
Neighbor-joining tree based on the ITS sequences obtained in this study (♦) and the related piroplasma. The ITS sequences of *B. rossi* were employed as an outgroup. GenBank accession numbers are indicated in parentheses. A bootstrap test of 1,000 replicates was used, and values are given at the nodes.

Phylogenetic analysis was performed to provide a better understanding of the diversity of the sequences. The neighbor-joining tree showed that all obtained 18S rRNA sequences clustered together with the 18S rRNA gene sequences of *B. gibsoni* and fell into the *Babesia* clade. Three large canine species (*B. vogeli, B. canis*, and *B. rossi*) fell into the same clade, while the rest of the canine species formed different clades (Figure 2). All ITS sequences obtained in this study also fell into the same group with *B. gibsoni* (Figure 3).

## Discussion

In this study, microscopic examination and RLB were used to detect canine piroplasmosis. RLB has been widely employed for *Babesia* and *Theileria* detection since this technique enables the simultaneous detection and discrimination of infections caused by these pathogens (Gubbels et al., 1999; Georges et al., 2001). This assay is very helpful for identifying new species and novel genotypes (Chaisi et al., 2011; Khan et al., 2013). Fourteen samples out of 118 (11.86%) were positive by RLB, and all positive samples were singly infected with *B. gibsoni*. The rate of positivity by RLB was higher than that obtained through microscopic examination because RLB is more sensitive.

Regarding canine babesiosis in China, Chen et al. (2014) identified *B. gibsoni* in two dogs in Henan. *B. gibsoni* is the most widespread species in China; it has been reported in Shandong, Jiangsu, Anhui, Shanghai, Zhejinag, Nanjing, Jiangxi, and Guangxi. *B. canis vogeli* is the other species reported in dogs, whose epidemic areas include Jiangxi and Gansu (Wei et al., 2012; Yao et al., 2014; Niu et al., 2017; Zheng et al., 2017). Very recently, Niu et al. reported *T. sinensis* in pet dogs in Gansu, representing the first report of *T. sinensis* in dogs (Niu et al., 2017). Previous studies have indicated that canine babesiosis is mainly epidemic in eastern and southern China, while limited information is available on its prevalence in central China. In this study, 118 samples were randomly collected from pet dogs and screened via RLB. Fourteen samples were hybridized with a species-specific probe for *B. gibsoni*. Sequence analysis of the 18S rRNA gene and the ITS regions showed that the obtained sequences shared high identity with the 18S rRNA and ITS genes of *B. gibsoni*, respectively. Phylogenetic trees based on the 18S rRNA gene and ITS region were generated. The results confirmed that all positive dogs exhibited single infection with *B. gibsoni*. We assume that *B. gibsoni* is the only species infecting dogs in Wuhan. The initial purpose of this study was to detect the occurrence of piroplasm in pet dogs, and only limited samples were collected and analyzed. An epidemiological investigation considering the dog population and characteristics such as age and breed should be performed as this study showed a high rate of positivity, of 11.86%. *B. gibsoni* was previously thought to be prevalent only in Asian countries (Irwin and Jefferies, 2004; Goo et al., 2008). However, it has spread to South Africa, America, Europe and many other areas around the world with notable speed (Birkenheuer et al., 2005; Matjila et al., 2007). The disease can spread through biting, fighting, and transplacental transmission rather than simply via ticks, which is why *B. gibsoni* has spread so quickly worldwide (Jefferies et al., 2007; Yeagley et al., 2009). As babesiosis is a tick-borne disease, its distribution normally depends on the prevalence of the ticks responsible for transmission. However, no ticks were found on the dogs sampled in this study. We infer that either the ticks had already dropped off of the dogs when they were taken to the clinic, or *B. gibsoni* was inherited from the dogs' parents or through activities such as dog fighting.

The clinical manifestations of dogs infected with *B. gibsoni* vary from subclinical to severe to even fatal, based on the physical condition of the host (Schnittger et al., 2012). Among the 14 positive dogs, four of the samples tested negative by microscopic examination. These four dogs exhibited subclinical infections without significant clinical symptoms. There will be a greater risk when these dogs are subjected to immunosuppressive conditions. On the other hand, regardless of whether significant clinic manifestations are present, infected dogs can become reservoirs and infect other *B. gibsoni*-free dogs.

In conclusion, this study provides the first molecular record from Wuhan, China using a molecular RLB assay to simultaneously detect canine piroplasmosis in pet dogs. *B. gibsoni* was the only identified species. The results showed a considerable rate of positivity in pet dogs. As pets are considered companion animals and play increasingly important roles in humans' lives, their health deserves greater attention. It is necessary to pay attention and monitor this disease in dogs.

## Author contributions

LH and XM wrote the draft of the manuscript. JZ and LH designed the study and corrected the manuscript. LH, XM, JiH, YH, PH, JuH, LY, NM, and LS collected samples and performed the molecular assays.

### Conflict of interest statement

The authors declare that the research was conducted in the absence of any commercial or financial relationships that could be construed as a potential conflict of interest.
